# Intergenerational educational mobility and intrinsic capacity in later life: a harmonised analysis of individuals from five countries

**DOI:** 10.7189/jogh.16.04304

**Published:** 2026-10-09

**Authors:** Lin Guo, Hongjing Wang, Wenjie Teng, Bingyu Bai, Bin Sang, Linlin Zhang

**Affiliations:** 1 School of Management, Shandong Second Medical University, Weifang, Shandong, China; 2 School of Basic Medical Sciences, Shandong Second Medical University, Weifang, Shandong, China; 3 School of Nursing, Shandong Second Medical University, Weifang, China; 4 School of Accounting, Zhejiang Financial College, Hangzhou, China

**Keywords:** intrinsic capacity, intergenerational educational mobility, healthy ageing, life course epidemiology, cross-national comparison, mediation analysis

## Abstract

**Background:**

The World Health Organization’s intrinsic capacity (IC) framework shifts focus from disease to functional ageing. Despite education’s role in health, the impact of intergenerational educational mobility (IEM) on IC trajectories remains understudied. We aimed to examine the cross-national association between IEM and IC, its effect on biological ageing rates, and underlying mechanisms.

**Methods:**

We analysed harmonised longitudinal data from five nationally representative cohorts: China Health and Retirement Longitudinal Study, Health and Retirement Study (USA), Survey of Health, Ageing and Retirement in Europe, Korean Longitudinal Study of Ageing, and Mexican Health and Aging Study, including 495,028 observations from 100,362 individuals aged ≥50. We standardised IC as a composite of cognition, locomotion, vitality, sensory, and psychological domains. We used a two-stage individual participant data meta-analysis, growth curve modelling, and the Karlson–Holm–Breen mediation method to assess associations, trajectories, and pathways *via* wealth, behaviours, and chronic conditions.

**Results:**

Upward IEM was robustly linked to higher IC (pooled *β* = 0.25; 95% confidence interval = 0.24, 0.26; *P* < 0.001). Linear mixed-effects models showed distinct intra-individual ageing patterns: upward mobility significantly decelerated the rate of IC decline (‘delayed ageing’) in China, Europe, South Korea, and Mexico. Conversely, a ‘parallel ageing’ pattern emerged in the USA, where mobility massively elevated baseline capacity but did not alter the slope of age-related decline.

**Conclusions:**

The IEM confers consistent health advantages across countries, but the magnitude and trajectory of these benefits are context-dependent. The unique ‘US exception’ of parallel ageing suggests that while mobility elevates early-life health capital, systemic environmental or healthcare factors may equalise subsequent late-life decline. Enhancing mobility remains a critical global strategy for healthy longevity.

Population ageing is a defining demographic trend of the 21st century, requiring a fundamental transformation of health systems worldwide. Historically, clinical practice has relied on a disease-centred paradigm that focuses on diagnosing and managing specific pathologies. However, this approach is increasingly insufficient for older populations, where multimorbidity is the norm. To address this limitation, the World Health Organization (WHO) introduced the construct of intrinsic capacity (IC), advocating for a paradigm shift toward a function-centred model of healthy ageing [1]. Defined as ‘the composite of all the physical and mental capacities that an individual can draw on at any point in time’, IC encompasses five pivotal domains: locomotion, vitality, cognition, psychological capacity, and sensory function [2,3]. Unlike frailty, which focuses on accumulated deficits and vulnerability, IC emphasises the positive physiological reserves an individual possesses, offering a more proactive, longitudinal metric to monitor functioning trajectories before disability sets in [4].

While IC provides a metric for healthy ageing, identifying its upstream determinants is crucial for intervention. Education is widely recognised as a fundamental ‘root cause’ of health; however, assessing an individual’s own educational attainment in isolation fails to capture the dynamic accumulation of advantage or disadvantage across the life course [5,6]. Intergenerational educational mobility (IEM) – the trajectory of educational attainment relative to one's parents – offers a more nuanced lens, integrating early-life conditions (parental capital) with adult achievement (personal capital) [7]. Two competing yet complementary theoretical frameworks elucidate how IEM shapes ageing trajectories. The Cumulative Advantage theory posits that upward IEM allows individuals to accumulate multidimensional resources – such as wealth, psychosocial buffers, and healthier environments – that progressively decelerate the rate of biological ageing [8,9]. Conversely, the Resource Substitution theory suggests that educational gains may yield greater marginal health returns for those from disadvantaged backgrounds, acting as a critical compensatory mechanism in the absence of other forms of capital [10].

Despite the theoretical plausibility of these frameworks, empirical evidence linking IEM to IC remains limited and fragmented. First, most existing literature comes from high-income countries, leaving a critical knowledge gap in middle-income contexts like China or Mexico, where rapid social transformations and distinct institutional arrangements may shape ageing differently [11,12]. Although recent multi-cohort studies have begun to explore life-course socioeconomic positions [5], systematic comparisons of mobility effects across diverse welfare regimes remain scarce. Second, it remains unclear whether upward mobility merely improves baseline health or effectively alters the velocity of biological ageing. While some evidence from the USA suggests mobility may slow biological ageing [9], whether this ‘delayed ageing’ pattern holds globally or varies by national context is unknown. Third, the mechanisms underlying this association remain poorly understood. While material pathways such as wealth accumulation are often emphasised, the relative contribution of non-material pathways – such as cognitive reserve or health behaviours – warrants rigorous quantification to inform targeted interventions.

To address these critical knowledge gaps, we leveraged harmonised longitudinal data from five nationally representative ageing cohorts: China Health and Retirement Longitudinal Study (CHARLS), Health and Retirement Study (HRS), Survey of Health, Ageing and Retirement in Europe (SHARE), Korean Longitudinal Study of Ageing (KLoSA), and Mexican Health and Aging Study (MHAS). Using a pooled sample of nearly 500,000 observations, we aimed to assess the cross-national association between IEM and IC, examining both the consistency and context-dependency of protective effects across diverse settings. Additionally, we aimed to determine whether IEM is associated with ‘parallel’ (intercept-only) or ‘delayed’ (slope-altering) ageing patterns using age-by-mobility interaction analyses, and to disentangle the direct and indirect pathways mediated by household wealth, health behaviours, and chronic conditions. By integrating diverse socio-political contexts, we sought to identify both consistent patterns and context-specific nuances to inform global healthy ageing policies.

## METHODS

### Data sources

We utilised harmonised longitudinal data from five nationally representative ageing cohorts: CHARLS, HRS, SHARE, KLoSA, and MHAS. The Peking University conducted CHARLS, and we used all five waves available in the harmonised dataset, spanning from 2011 to 2020. National Institute on Aging sponsored the HRS, and we used 11 biennial waves from 2000 (wave 5) to 2020 (wave 15), since early waves lacked consistent physical performance measures. Furthermore, the Munich Center for the Economics of Aging coordinated the SHARE, and we utilised the data from 7 waves (waves 1, 2, and 4–8) collected between 2004 and 2020, excluding Wave 3 (SHARELIFE) as it was retrospective. The Korea Employment Information Service conducted KLoSA, and we included six waves spanning from 2010 (wave 3) to 2020 (wave 8) because of the availability of harmonised IC domains. Lastly, the National Institute of Statistics and Geography and the UTMB sponsored MHAS, and we restricted our analysis to three continuous waves fielded in 2012, 2015, and 2018 (waves 3–5) to ensure covariate completeness.

We obtained the original data sets from each study's official repository. To ensure rigorous cross-national comparability, we employed the Harmonized Data Files developed by the Gateway to Global Aging Data [13], where variables were systematically cleaned and standardised across cohorts. All surveys are part of the global family of Health and Retirement Studies and were conducted in accordance with the Declaration of Helsinki.

### Study population and analytic sample

Based on the selected waves, the initial pooled data set comprised 753,347 observations from CHARLS (n = 96,628), HRS (n = 208,674), SHARE (n = 327,266), KLoSA (n = 44,273), and MHAS (n = 76,506). To ensure a robust analysis of ageing trajectories, we applied a sequential exclusion process (Table S1 in the **Online Supplementary Document**).

First, we excluded 54,861 observations with respondents aged <50 years or missing age information. Second, we removed 8825 observations with missing data on the core dependent variable (IC indicators). Third, we excluded 114,198 observations with missing IEM information (own or parental education). Finally, to ensure complete-case analysis, we removed 80,435 observations with missing covariate values, including demographic characteristics, socioeconomic status, lifestyle behaviours, and baseline chronic conditions.

To assess potential selection bias from excluding observations with missing IEM data, we conducted a supplementary sensitivity analysis comparing the demographic and health characteristics of the excluded 114,198 observations with the 495,028 included observations (Table S3 in the **Online Supplementary Document**). Excluded participants were, on average, older (mean (x̄) age difference = 4.36 years; *P* < 0.001), had lower IC scores (x̄ difference = −0.22; *P* < 0.001), and were disproportionately from the CHARLS and MHAS cohorts. These patterns suggest that the analytic sample is positively selected on health and socioeconomic status, and individuals in the stable low group are likely underrepresented. This would bias estimates toward the null, making our reported associations conservative.

The final analytic sample consisted of 495,028 observations from 100,362 unique individuals from CHARLS (n = 53,853), HRS (n = 167,184), SHARE (n = 193,767), KLoSA (n = 42,829), and MHAS (n = 37,395).

### Measures

All variables were harmonised across the five cohorts following the protocols of the Gateway to Global Aging Data to ensure cross-national comparability (Table S2 in the **Online Supplementary Document**).

### Outcome: IC

The primary outcome was IC, constructed as a standardised composite score based on the WHO framework. Following established methodologies, IC encompasses five functional domains: cognition, locomotion, vitality, sensory function, and psychological well-being. We calculated Z-scores for each domain within each cohort and wave, averaged them to create a global score, and then re-standardised the final score (x̄ = 0; standard deviation (SD) = 1). Higher scores indicate better IC.

### Exposure: IEM

The IEM tracks the educational trajectory from parents to offspring. We harmonised educational attainment into a binary metric (‘low’ *vs.* ‘high’) based on the ISCED-97 classification, with upper secondary education as the primary threshold. By cross-classifying parental and offspring education, we categorised participants into four trajectories: stable low (reference), upward IEM, stable high, and downward IEM.

### Covariates

We adjusted for potential confounders, including demographic factors (age, sex, marital status), socioeconomic status (household wealth tertiles, employment status), health behaviours (smoking status), and baseline health (count of seven major chronic conditions). The seven major chronic conditions – hypertension, diabetes, cancer, lung disease, heart disease, stroke, and arthritis – were selected because they represent the most prevalent chronic conditions in ageing populations globally [1] and are consistently assessed across all five cohorts in the harmonised data set, ensuring cross-national comparability.

### Statistical analysis

#### Cross-national association and meta-analysis

To assess the comprehensive impact of IEM on IC, we adopted a robust two-stage individual participant data meta-analysis strategy. In the first stage, multivariable linear regression models were fitted separately for each of the five cohorts to estimate cohort-specific standardised coefficients (*β*) for IEM groups, using ‘stable low’ as the reference. These models incorporated survey-wave fixed effects to adjust for secular trends and temporal variation, and estimated cluster-robust standard errors at the individual level to account for the non-independence of repeated measures. In the second stage, given anticipated contextual heterogeneity across countries, we synthesised these cohort-specific estimates using a random-effects meta-analysis model that accounts for both within-study sampling error and between-study variance. We further quantified heterogeneity using the *I*^2^ statistic and Cochran’s Q test. Given the extremely high heterogeneity observed (*I*^2 ^> 98%), we present both pooled estimates and country-specific effects, emphasising the context-dependent nature of the IEM–IC association.

#### Longitudinal trajectories of ageing

To determine whether educational mobility alters the rate of biological ageing (slope) rather than merely the baseline health (intercept), we applied growth curve modelling principles. We constructed linear regression models incorporating an interaction term between mobility groups and age (mobility × age). We centred age at 60 years to ensure the intercept represented health status at a meaningful baseline age. A significant positive interaction coefficient indicates a ‘delayed ageing’ pattern (slower decline), whereas a non-significant interaction suggests ‘parallel ageing’.

#### Mechanism decomposition

To disentangle the pathways linking IEM to IC, we employed the Karlson–Holm–Breen (KHB) method. This approach decomposes the total effect into direct effects (unmediated benefits) and indirect effects mediated by material resources (household wealth), health behaviours (smoking), and baseline health (chronic conditions). We calculated the confounding percentage to quantify each mediator's relative contribution.

#### Subgroup and sensitivity analyses

We conducted extensive robustness checks on the pooled data set. First, we stratified analyses by age, sex, wealth, and employment status to examine effect heterogeneity. Second, to address potential biases, we conducted three validation tests: excluding participants with fewer than three follow-up waves to mitigate attrition bias; excluding individuals with multiple baseline chronic conditions to minimise reverse causality; and employing propensity score matching (PSM) to estimate the average treatment effect on the treated, thereby ensuring that the observed benefits were not driven by baseline demographic imbalances.

We performed all statistical analyses using Stata, version 17.0 (StataCorp LLC, College Station, Texas, USA). Statistical significance was defined as a two-sided *P*-value <0.05.

## RESULTS

### Baseline characteristics of the study populations

We included 495,028 observations in the final analysis (**Table 1**). The x̄ age ranged from 62.69 years (SD = 8.76) in CHARLS to 67.97 years (SD = 10.3) in KLoSA. The standardised IC scores were relatively consistent across populations, with HRS showing the highest average (x̄ = 0.09; SD = 0.96). Regarding health status, individuals in HRS reported the highest burden of chronic conditions (x̄ = 1.87), while those in KLoSA reported the lowest (x̄ = 1.09).

**Table 1:** Baseline characteristics of participants across cohorts*

	CHARLS	HRS	SHARE	KLoSA	MHAS
**IC, x̄ (SD)†**	−0.06 (0.99)	0.09 (0.96)	0.03 (0.98)	−0.01 (1)	0 (1)
**Age (years), x̄ (SD)**	62.69 (8.76)	65.99 (9.37)	66.49 (9.9)	67.97 (10.3)	65.18 (9.86)
**Chronic conditions, x̄ (SD)**	1.25 (1.15)	1.87 (1.37)	1.32 (1.23)	1.09 (1.11)	1.32 (1.15)
**IEM**					
Stable low	45,770 (84.99)	22,825 (13.65)	64,520 (33.3)	24,522 (57.26)	29,890 (79.93)
Upward	5,829 (10.82)	49,355 (29.52)	55,889 (28.84)	15,714 (36.69)	4,050 (10.83)
Stable high	796 (1.48)	5,953 (3.56)	61,803 (31.9)	2,190 (5.11)	1,873 (5.01)
Downward	1,458 (2.71)	89,051 (53.27)	11,555 (5.96)	403 (0.94)	1,582 (4.23)
**Sex**					
Female	28,171 (52.31)	95,315 (57.01)	108,952 (56.23)	24,637 (57.52)	21,745 (58.15)
Male	25,682 (47.69)	71,869 (42.99)	84,815 (43.77)	18,192 (42.48)	15,650 (41.85)
**Marital status**					
Others	7,466 (13.86)	62,464 (37.36)	56,894 (29.36)	10,385 (24.25)	12,021 (32.15)
Married/partnered	46,387 (86.14)	104,720 (62.64)	136,873 (70.64)	32,444 (75.75)	25,374 (67.85)
**Household wealth tertiles‡**					
Bottom	18,192 (33.78)	56,406 (33.74)	64,589 (33.33)	14,520 (33.9)	12,467 (33.34)
Middle	17,775 (33.01)	55,153 (32.99)	64,589 (33.33)	14,044 (32.79)	12,463 (33.33)
Top	17,886 (33.21)	55,625 (33.27)	64,589 (33.33)	14,265 (33.31)	12,465 (33.33)
**Employment status**					
Not working	19,930 (37.01)	98,332 (58.82)	128,830 (66.49)	25,891 (60.45)	22,818 (61.02)
Currently working	33,923 (62.99)	68,852 (41.18)	64,937 (33.51)	16,938 (39.55)	14,577 (38.98)
**Smoking status**					
Non-smoker	39,290 (72.96)	143,294 (85.71)	160,561 (82.86)	37,441 (87.42)	33,029 (88.32)
Current smoker	14,563 (27.04)	23,890 (14.29)	33,206 (17.14)	5,388 (12.58)	4,366 (11.68)

CHARLS – China Health and Retirement Longitudinal Study, HRS – Health and Retirement Study, IC – intrinsic capacity, IEM – intergenerational educational mobility, KLoSA – Korean Longitudinal Study of Ageing, MHAS – Mexican Health and Aging Study, SD – standard deviation, SHARE – Survey of Health, Ageing and Retirement in Europe, x̄ – mean

*Values are presented as n (%) unless specified otherwise.

†Intrinsic Capacity scores were standardised (Z-scores) within each cohort to ensure comparability.

‡Household wealth tertiles were calculated based on cohort-specific total household assets.

The IEM patterns varied significantly across countries. In developing contexts, most participants remained in the stable low category, particularly in CHARLS (84.99%) and MHAS (79.93%), reflecting limited educational opportunities for older generations in these regions. In contrast, the upward IEM group was most prominent in KLoSA (36.69%), while HRS showed a uniquely high proportion of the downward IEM group (53.27%).

Demographic and lifestyle factors also varied. The CHARLS cohort had the highest proportions of married individuals (86.14%), currently working participants (62.99%), and current smokers (27.04%). In terms of gender distribution, all cohorts showed a slight female predominance, ranging from 52.31% in CHARLS to 58.15% in MHAS. We evenly distributed household wealth into tertiles across all populations by design.

To assess potential multicollinearity, we examined bivariate associations among all variables using Spearman’s rank correlation. The correlation coefficients between covariates were generally low to moderate (Figure S1 in the **Online Supplementary Document**). The strongest correlation was observed between age and work status (*r* = −0.50), which is expected given retirement patterns. All correlation coefficients were well below the conventional threshold of 0.7, indicating that multicollinearity was not a concern in this study.

### Association between IEM and IC

Mobility affected IC substantially differently across welfare regimes (**Figure 1**). Notably, upward and stable high mobility show the strongest positive effects in the Chinese cohort (CHARLS). In contrast, the downward mobility trajectory appears to play a more prominent role in the USA (HRS), suggesting distinct patterns of health inequality across these contexts.

**Figure 1:**
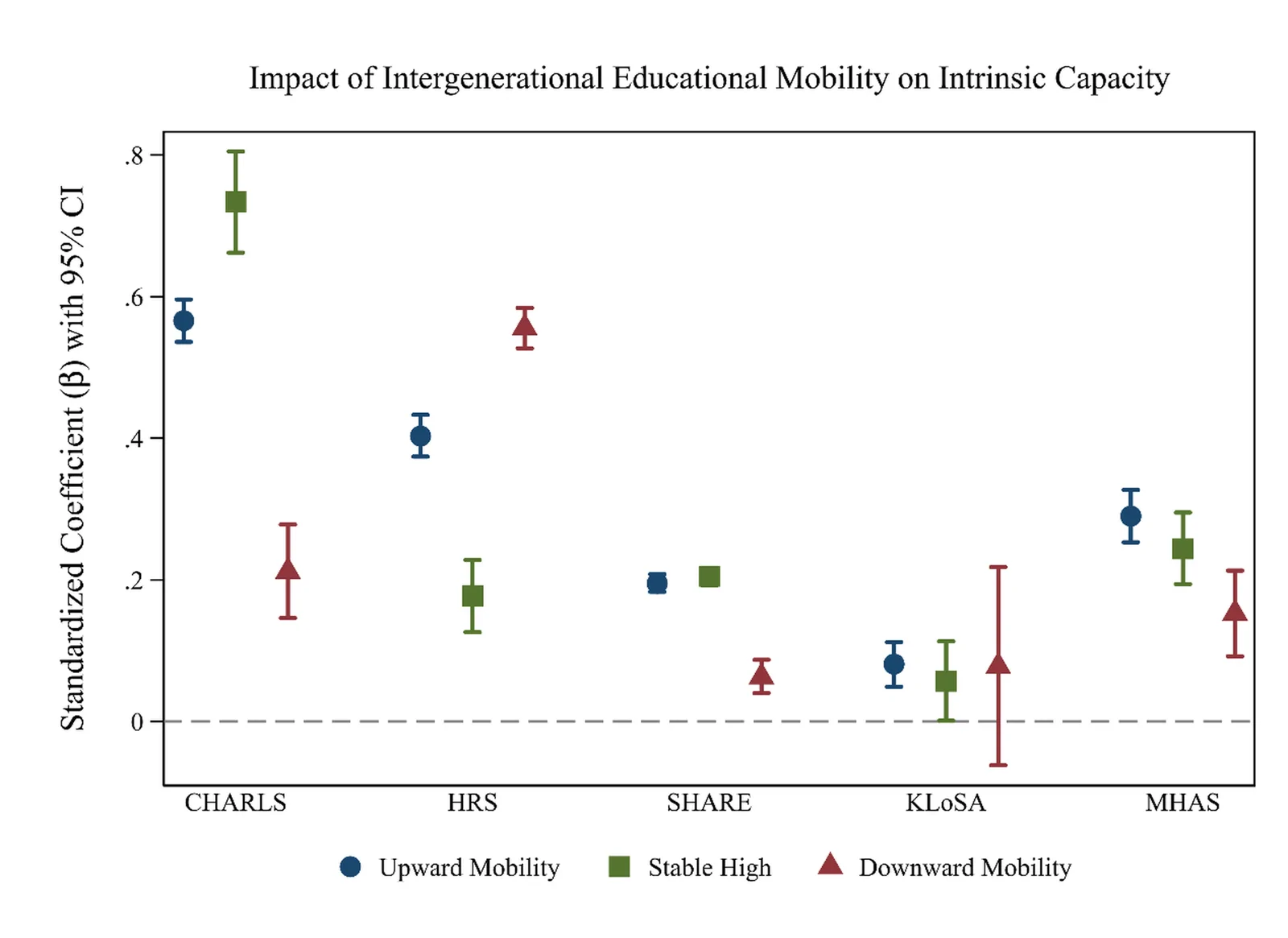
Impact of intergenerational educational mobility on intrinsic capacity. CHARLS – China Health and Retirement Longitudinal Study, HRS – Health and Retirement Study, KLoSA – Korean Longitudinal Study of Ageing, MHAS – Mexican Health and Aging Study, SHARE – Survey of Health, Ageing and Retirement in Europe.

### Overall pooled effects

The meta-analysis found that all three IEM trajectories were associated with better IC than the stable low group. Specifically, individuals who experienced upward IEM demonstrated significantly higher IC (pooled *β* = 0.25; 95% confidence interval (CI) = 0.24, 0.26; *P* < 0.001). Similarly, the stable high group showed a positive effect (pooled *β* = 0.21; 95% CI = 0.20, 0.22; *P* < 0.001). Notably, even individuals in the downward mobility trajectory exhibited better IC performance than the reference group (pooled *β* = 0.25; 95% CI = 0.23, 0.27; *P* < 0.001) (Figure S2 in the **Online Supplementary Document**).

### Heterogeneity and cohort-specific findings

Substantial heterogeneity was observed across countries in all three IEM categories (*I*^2 ^> 98%, *P* < 0.001), which constitutes a central finding of this study. The consistency in the direction of effects (all mobility groups show higher IC than stable low in all countries) is notable, but the magnitude of the association varies dramatically across contexts.

### Upward IEM

The health return on upward IEM was most pronounced in China (CHARLS) (*β* = 0.57; 95% CI = 0.54, 0.60), followed by the USA (HRS) (*β* = 0.40) and Mexico (MHAS) (*β* = 0.29). In contrast, the effect sizes were more modest in Europe (SHARE) (*β* = 0.19) and lowest in South Korea (KLoSA) (*β* = 0.08). This nearly 7-fold difference between the strongest and weakest effects underscores the context-dependent nature of the IEM–IC relationship.

### Stable high

China (CHARLS) exhibited an exceptionally large effect size for the stable high group (*β* = 0.73; 95% CI = 0.66, 0.81), suggesting a strong cumulative advantage of high intergenerational education in the Chinese context. Other cohorts showed moderate positive effects ranging from 0.18 to 0.24, except for South Korea, where the effect was minimal (*β* = 0.06).

### Downward mobility

Interestingly, the protective effect of downward mobility (having highly educated parents despite low personal education) was strongest in the USA (HRS) (*β* = 0.56; 95% CI = 0.53, 0.58), surpassing even the upward mobility effect in the same cohort. Conversely, in South Korea (KLoSA), downward mobility was not significantly associated with IC (*β* = 0.08; 95% CI = −0.06, 0.22), as the CI crossed zero.

### Longitudinal trajectories of IC decline

To determine whether IEM alters the true intra-individual rate of biological ageing, we fitted formal linear mixed-effects models with random intercepts at the individual level and age-by-mobility fixed interaction terms (**Figure 2**, Panels A–F).

**Figure 2:**
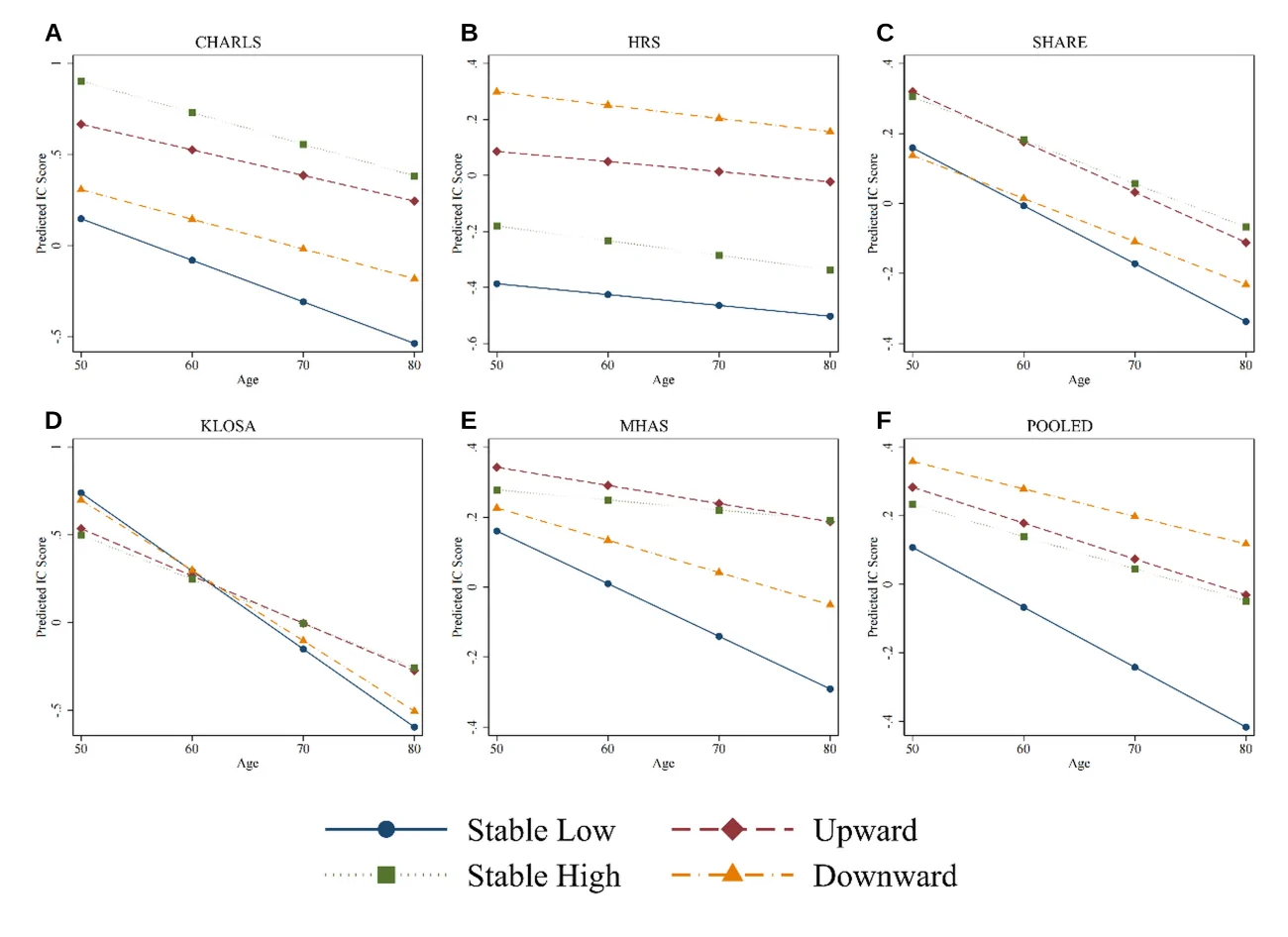
Predicted longitudinal trajectories of IC decline by IEM. **Panel A.** CHARLS. **Panel B.** HRS. **Panel C.** SHARE. **Panel D.** KLoSA. **Panel E.** MHAS. **Panel F.** Pooled. Models are adjusted for sex, marital status, household wealth, smoking, employment, chronic conditions, and survey waves. CHARLS – China Health and Retirement Longitudinal Study, HRS – Health and Retirement Study, IC – intrinsic capacity, IEM – intergenerational educational mobility, KLoSA – Korean Longitudinal Study of Ageing, MHAS – Mexican Health and Aging Study, SHARE – Survey of Health, Ageing and Retirement in Europe.

### Global patterns of delayed ageing

The pooled analysis of 495,028 observations revealed a significant ‘cumulative advantage’ mechanism globally. The interaction between upward mobility and age was significantly positive (*β*_interaction_ = 0.007; *P* < 0.001), indicating that individuals experiencing upward mobility exhibited a slower rate of IC decline compared to the stable low group. Similarly, the downward mobility (*β*_interaction_ = 0.009; *P* < 0.001) and stable high (*β*_interaction_ = 0.008; *P* < 0.001) trajectories also demonstrated flatter slopes of decline. This confirms that, across diverse societies, exposure to high parental or personal education acts as a biological buffer against accelerated functional loss in later life.

### Cohort-specific heterogeneity: diverging *vs.* parallel trajectories

Stratified mixed-effects analyses uncovered distinct ageing dynamics across different national contexts:

We observed significant slope differences in most cohorts, including China (*β*_interaction_ = 0.008; *P* < 0.001), Europe (*β*_interaction_ = 0.002; *P* < 0.001), South Korea (*β*_interaction_ = 0.017; *P* < 0.001), and Mexico (*β*_interaction_ = 0.009; *P* < 0.001). In these contexts, the health gap between the upward mobility and stable low groups widens with advancing age. This ‘fanning out’ pattern indicates that educational mobility effectively reduces the velocity of functional deterioration over time.

Interestingly, a distinct pattern emerged in the USA. While upward mobility provided a massive initial advantage in IC at age 60 (*β* = 0.47; *P* < 0.001), the rate of decline did not differ significantly from the stable low group (*β*_interaction_ = 0.0002; *P* = 0.757). The trajectories remained strictly parallel (**Figure 2**, Panel B). This suggests that in the USA context, intergenerational educational advantages manifest primarily as a permanent elevation in baseline health (intercept) rather than a continuously decelerating rate of ageing.

### Disentangling the mechanisms: wealth as the primary mediator

Using the KHB method to disentangle the contribution of specific mediators, we found distinct pathways for different mobility trajectories (**Table 2**).

**Table 2:** Decomposition of the total effect of IEM on IC into direct and indirect effects*

	Upward mobility			Stable high			Downward mobility		
	***β* (95% CI)**	**SE**	**% total effect**	***β* (95% CI)**	**SE**	**% total effect**	***β* (95% CI)**	**SE**	**% total effect**
**Total effect**	0.399 (0.388, 0.409)	0.005	100.00	0.383 (0.371, 0.394)	0.006	100.00	0.487 (0.476, 0.499)	0.006	100.00
**Direct effect**	0.347 (0.337, 0.358)	0.005	87.09	0.290 (0.278, 0.301)	0.006	75.64	0.459 (0.447, 0.471)	0.006	94.20
**Indirect effect (total)**	0.051 (0.044, 0.059)	0.004	12.91	0.093 (0.086, 0.100)	0.004	24.36	0.028 (0.021, 0.035)	0.004	5.80
***Via* household wealth†**			10.40			11.20			11.11
***Via* chronic conditions**			2.60			13.01			−5.16
***Via* smoking status**			−0.08			0.15			−0.15

CI – confidence interval, IC – intrinsic capacity, IEM – intergenerational educational mobility, SE – standard error

*Estimates are derived from the Karlson-Holm-Breen method based on the pooled sample (n = 495,028); the ‘stable low’ group served as the reference category; all models were adjusted for age, sex, marital status, employment, and survey waves.

†The contribution of household wealth represents the aggregated effect of wealth tertiles.

### Wealth accumulation drives the benefit

Household wealth, particularly attaining the highest wealth tertile, emerged as the most consistent mediator. For the upward IEM group, high wealth explained 10.40% of the total effect on IC, suggesting that economic returns to education are a key pathway for health improvement. Similarly, high wealth accounted for an 11.20% advantage in the stable high group and an 11.11% advantage in the downward IEM group.

### The ‘disease accumulation’ pathway in stable elites

A striking disparity was observed in the role of chronic conditions. For the stable high group, reduced burden of chronic diseases explained 13.01% of their health advantage. In contrast, for the upward IEM group, chronic conditions only accounted for 2.60% of the effect. This indicates that intergenerational transmission of high education confers a long-term protection against disease onset (‘primary prevention’), whereas upward IEM improves functional ability mainly through mechanisms other than disease avoidance (*e.g.* better coping or management).

### Behavioural factors

Smoking status played a negligible role across all groups (<1% contribution), suggesting that the link between IEM and IC is not primarily driven by the avoidance of smoking, but rather by broader socioeconomic and biological pathways.

### Subgroup analyses

To identify potential effect modifiers and explore heterogeneity in the association, we conducted stratified analyses based on the pooled sample of 495,028 individuals (**Figure 3**, Panel A–C; **Table 3**). We consistently observed a positive association between IEM and IC across all subgroups (*P* < 0.001), though the effect size varied by sociodemographic characteristics.

**Figure 3:**
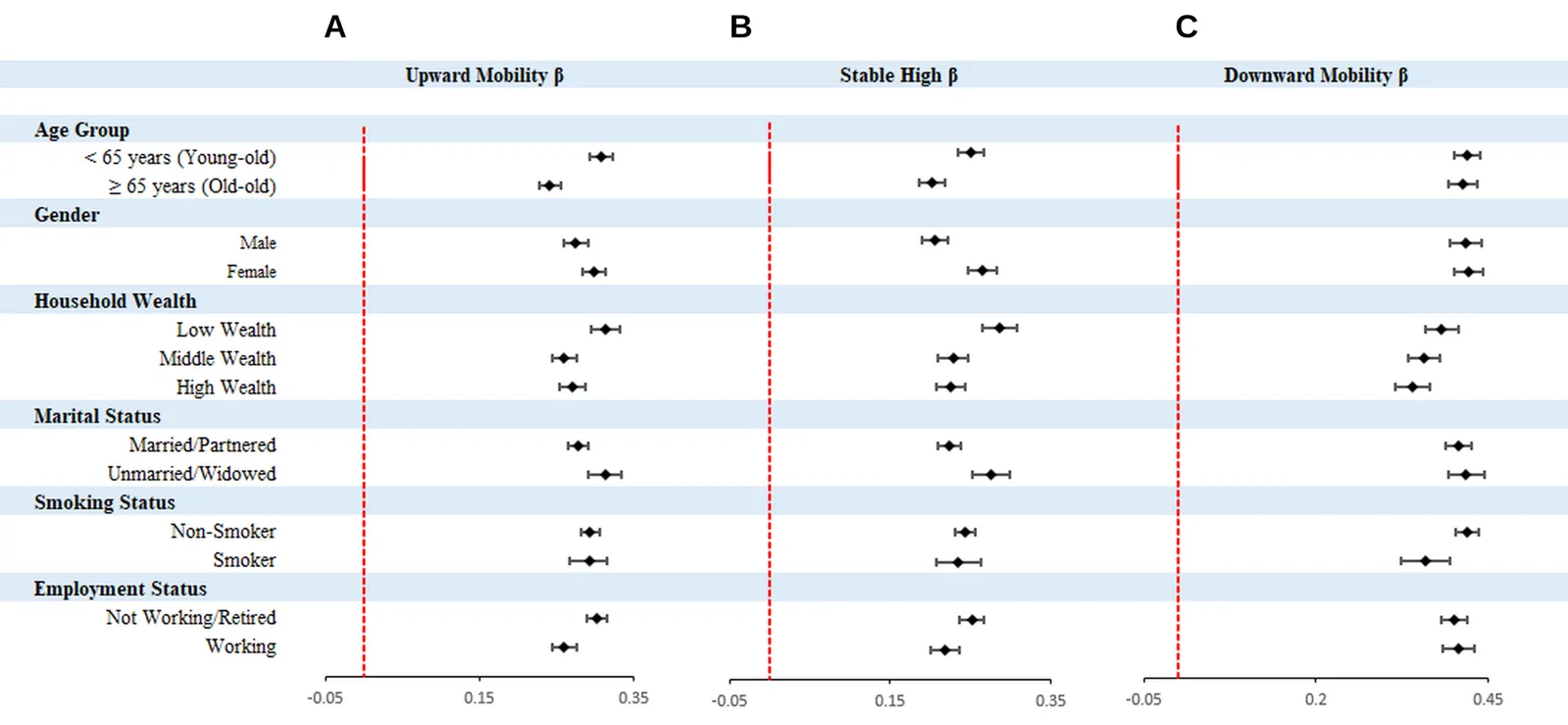
Subgroup analyses. **Panel A.** Upward mobility. **Panel B.** Stable high. **Panel C.** Downward mobility. The ‘stable low’ group served as the reference category; all models were adjusted for age (centred at 60), sex, marital status, household wealth tertiles, smoking status, employment status, chronic conditions, survey waves, and country dummies (variables used for stratification were excluded from their respective models); values are presented as *β* with 95% confidence intervals.

**Table 3:** Subgroup analyses of the association between IEM and IC*

		*β* (95% CI)		
	**n**	**Upward mobility**	**Stable high**	**Downward mobility**
**Age group in years**				
<65	239,080	0.241 (0.227, 0.255)	0.202 (0.186, 0.218)	0.414 (0.393, 0.434)
≥65	255,948	0.308 (0.293, 0.323)	0.250 (0.234, 0.267)	0.420 (0.400, 0.439)
**Gender**				
Male	278,820	0.299 (0.284, 0.314)	0.265 (0.249, 0.282)	0.421 (0.401, 0.442)
Female	216,208	0.275 (0.259, 0.290)	0.205 (0.187, 0.223)	0.417 (0.394, 0.440)
**Household wealth**				
Low	166,174	0.313 (0.295, 0.332)	0.286 (0.265, 0.307)	0.382 (0.358, 0.406)
Middle	164,024	0.260 (0.245, 0.276)	0.228 (0.209, 0.246)	0.356 (0.334, 0.379)
High	164,830	0.271 (0.254, 0.288)	0.225 (0.207, 0.244)	0.340 (0.314, 0.366)
**Marital status**				
Married/partnered	345,798	0.277 (0.265, 0.290)	0.224 (0.210, 0.237)	0.406 (0.388, 0.425)
Unmarried/widowed	149,230	0.313 (0.291, 0.334)	0.275 (0.252, 0.299)	0.418 (0.392, 0.444)
**Smoking status**				
Non-smoker	413,615	0.293 (0.281, 0.305)	0.243 (0.230, 0.256)	0.420 (0.403, 0.437)
Smoker	81,413	0.292 (0.267, 0.316)	0.235 (0.207, 0.263)	0.358 (0.323, 0.394)
**Employment status**				
Not working/retired	295,801	0.303 (0.289, 0.316)	0.252 (0.236, 0.267)	0.401 (0.382, 0.420)
Working	199,227	0.260 (0.244, 0.276)	0.218 (0.200, 0.236)	0.407 (0.384, 0.429)

CI – confidence interval, IC – intrinsic capacity, IEM – intergenerational educational mobility

*The ‘stable low’ group served as the reference category; all models were adjusted for age (centred at 60), sex, marital status, household wealth tertiles, smoking status, employment status, chronic conditions, survey waves, and country dummies (variables used for stratification were excluded from their respective models).

### Resource substitution: stronger benefits for the disadvantaged

Consistent with the ‘Resource Substitution’ theory, the health returns to upward IEM were more pronounced among socially vulnerable groups. Individuals in the low wealth tertile derived greater benefits from upward IEM (*β* = 0.313; 95% CI = 0.295, 0.332) compared to those in the high wealth tertile (*β* = 0.277; 95% CI = 0.265, 0.290). Similarly, unmarried or widowed individuals exhibited a stronger association (*β* = 0.313) than their married counterparts (*β* = 0.277). This suggests that without alternative resources (*e.g.* financial capital or spousal support), education serves as a critical compensatory mechanism for maintaining health.

### Cumulative advantage: age and employment dynamics

The protective effect of education appeared to accumulate over the life course. The standardised coefficient for upward IEM was substantially higher in the ≥65 years group (*β* = 0.308) than in the <65 years group (*β* = 0.241), indicating that the IEM-driven health gap widens with age. Furthermore, retired or non-working individuals showed a stronger association (*β* = 0.303) than those currently employed (*β* = 0.260), suggesting that the ‘cognitive reserve’ and health literacy provided by education become particularly vital for health maintenance after individuals exit the workforce.

### Gender and lifestyle stability

Regarding gender, males experienced a slightly higher health return from upward IEM (*β* = 0.299; 95% CI = 0.284, 0.314) compared to females (*β* = 0.275; 95% CI = 0.259, 0.290), though the difference was modest. This suggests that while education is consistently protective, men in these cohorts might have had greater opportunities to translate educational gains into occupational status and subsequent health resources. Notably, the association remained robust regardless of smoking status, with nearly identical effect sizes for non-smokers (*β* = 0.293) and smokers (*β* = 0.292), reinforcing that the neuroprotective and functional benefits of education operate independently of this specific behavioural risk factor.

### Sensitivity analyses

To test the robustness of our findings to potential biases, we conducted three sensitivity analyses. First, to mitigate attrition bias, we restricted the sample to participants with at least three waves of follow-up data (n = 420,887). The positive association between upward IEM and IC remained robust and even increased in magnitude (*β* = 0.317; 95% CI = 0.304, 0.330). Second, to address health selection bias and reverse causality, we excluded individuals with multiple chronic conditions (>1) at baseline. Even among this relatively healthy sub-sample (n = 363,856), IEM continued to show a significant protective effect (*β* = 0.288; 95% CI = 0.276, 0.301). Finally, we used PSM to balance demographic characteristics between the upward IEM and stable low groups. The average treatment effect on the treated confirmed a substantial and significant advantage for the upward IEM group (difference = 0.383, *t* = 89.98), providing strong evidence that the observed health benefits are not driven by confounding demographic factors.

## DISCUSSION

To our knowledge, this is the first study to leverage harmonised data from five national cohorts (n = 495,028) to comprehensively examine the association between IEM and IC across the life course. Our findings indicate that IEM is consistently associated with better IC across diverse national settings, supporting the value of educational equity policies for healthy ageing. However, the substantial cross-national heterogeneity (*I*^2 ^> 98%) indicates that the magnitude of this benefit is highly context-dependent, shaped by country-specific institutional, economic, and cultural factors. Specifically, we identified three key insights. First, while upward mobility was consistently associated with higher IC globally, the magnitude of the benefit varied significantly, with the strongest effects in China (CHARLS) and the weakest in South Korea (KLoSA). Second, we observed a distinct ‘Chinese Exception’: whereas educational mobility delayed the rate of ageing (slope) in high-income contexts (USA, Europe, South Korea), it primarily elevated baseline health (intercept) in China, resulting in parallel ageing trajectories. Third, mobility’s protective effect was predominantly direct (87%), likely reflecting cognitive reserve, though wealth accumulation played a critical mediating role (11–14%), supporting the materialist pathway.

### Contextualising divergent ageing trajectories

Our linear mixed-effects models revealed an unexpected but theoretically profound divergence: while upward mobility delayed the velocity of ageing in China, Europe, South Korea, and Mexico, it manifested strictly as ‘parallel ageing’ in the USA.

The parallel trajectory in the USA strongly aligns with the ‘age-as-leveller’ hypothesis [14]. Our data show that while upward mobility grants a large initial health advantage at age 60 (a large intercept difference), the subsequent rate of decline is statistically indistinguishable from that of those remaining in the lower class. This equalisation may be driven by systemic US-specific factors. For instance, universal Medicare eligibility at age 65 provides a safety net that might narrow late-life healthcare disparities. Furthermore, the pervasive exposure to obesogenic environments and cardiometabolic risks in the USA may eventually override the protective buffers of education, causing biological senescence to proceed at a uniform rate regardless of class origins [15].

Conversely, the robust ‘delayed ageing’ (diverging slopes) observed in China and other rapidly transforming or welfare-state contexts supports the ‘Cumulative Advantage’ theory [8]. In China, the initial health resources and literacy gained through early-life educational mobility continue to compound. In a rapidly developing society where premium medical resources and eldercare are highly stratified by socioeconomic status [16], the upwardly mobile can leverage these ongoing advantages to slow functional decline, widening the health gap as they age.

### Disentangling mechanisms: cognitive reserve *vs.* material wealth

Our mediation analysis *via* the KHB method offers a nuanced understanding of how mobility gets ‘under the skin’. We found that household wealth was the strongest mediator (explaining 11–14% of the effect), supporting the materialist perspective that economic resources are pivotal for healthy ageing [15,17]. Wealth affords better nutrition, housing, and medical care, which directly sustain IC [5].

However, the fact that nearly 87% of the effect remained direct highlights the critical role of non-material pathways. This large residual effect likely points to ‘cognitive reserve’ [18] and ‘health literacy’. Education enhances neural plasticity and compensatory networks, allowing individuals to maintain higher functional performance (IC) despite accumulating neuropathology [19]. Additionally, the ‘direct’ benefit may reflect early-life biological embedding. Our finding that downward mobility (high parental education but low personal education) still conferred a significant health advantage (*β* = 0.42) suggests that the benefits of parental human capital – transmitted through genetics, early nutrition, or parenting – persist biologically regardless of the offspring's adult status [20,21].

Interestingly, we found that smoking mediated a negligible portion (<1%) of the association. This challenges the common assumption that health behaviours are the primary driver of educational gradients [22], suggesting that structural and biological mechanisms (*e.g.* allostatic load, inflammation) may be more salient targets for intervention than individual lifestyle choices alone [6].

An important nuance warranting consideration is the potential role of educational field, beyond educational level, in shaping later-life IC. Individuals with training in health-related or scientific disciplines may acquire greater health literacy, which could translate into more effective health information seeking, better self-management of chronic conditions, and more proactive engagement with healthcare systems [23]. Conversely, the general cognitive skills and critical thinking cultivated across many disciplines may also confer broad protective benefits [24]. The harmonised data used in this study do not contain information on field of study; future research incorporating detailed educational specialisation data could disentangle whether certain fields amplify the protective effects of IEM on functional ageing, thereby informing more targeted educational and public health interventions.

The benefits of higher parental education may extend beyond offspring's formal educational attainment. Parents with higher education may transmit health-promoting knowledge, attitudes, and behaviours – such as dietary practices, health-seeking behaviours, and cognitive stimulation – through informal channels within the family environment [25,26]. This 'cultural capital' transmission may partly explain why downward IEM (having highly educated parents despite low personal education) still confers a protective effect on IC in several countries, as observed in our results. The particularly strong protective effect of downward IEM in the USA (*β* = 0.56) is consistent with this interpretation, as it suggests that the benefits of parental education persist even when offspring do not achieve high educational attainment themselves. While the harmonised cohort data used in this study do not permit direct measurement of such informal knowledge transfer, future qualitative and mixed-methods research could illuminate these intergenerational mechanisms.

### Subgroup heterogeneity: resource substitution and status maintenance

Our subgroup analyses revealed two intriguing patterns. First, consistent with the ‘Resource Substitution’ theory [10], the health returns to upward mobility were significantly stronger among the resource-poor (*β* = 0.313) than the resource-rich (*β* = 0.277). For individuals lacking economic capital, education acts as a critical compensatory resource – a ‘lifeline’ that substitutes for the absence of material wealth to protect health [27].

Second, regarding gender, we observed a slight male advantage (*β* = 0.299 *vs.* *β* = 0.275). While some studies suggest women benefit more from education [28,29], our results align with the status maintenance perspective [30]. In the historical context of these older cohorts, men were often the primary breadwinners and had greater opportunities to convert educational credentials into occupational prestige, social networks, and authority – resources that robustly support IC [31]. Conversely, women’s educational gains might have been constrained by labour market discrimination or interrupted by caregiving roles, slightly dampening the health conversion rate [32].

### Strengths and limitations

Strengths of this study include large-scale, harmonised multinational data (n = 495,028), the application of the novel WHO IC framework [2,3], and rigorous causal inference techniques (PSM, KHB).

However, limitations remain. First, differences in measurement modalities for IC domains across cohorts warrant explicit acknowledgement. Specifically, KLoSA relied on a self-reported measure of locomotion (difficulty climbing stairs) rather than objective physical performance tests, and SHARE employed animal naming (verbal fluency) rather than the Serial 7s subtraction test used in other cohorts for the mental status component. Additionally, depressive symptoms were assessed using the CES-D scale in four cohorts but the Euro-D scale in SHARE. While we employed within-cohort Z-score standardisation and domain-level averaging to mitigate these differences, we acknowledge that measurement non-equivalence may introduce residual variability. The IC composite has been validated as a robust predictor of adverse outcomes across diverse settings [29], supporting its construct validity despite measurement heterogeneity. Nonetheless, future harmonisation efforts should prioritise standardised IC assessment protocols across international ageing studies.

Second, we utilised a binary threshold for educational mobility to maximise comparability across heterogeneous global education systems. This prevented us from capturing the dose-response magnitude of educational mobility (*e.g.* distinguishing the health returns of advancing from primary to secondary *vs.* primary to tertiary education), which warrants future granular investigation.

In addition, excluding 114,198 observations due to missing IEM data raises important concerns about selection bias, suggesting that the stable low reference group is likely underrepresented in the analytic sample and would tend to bias our estimates of the IEM–IC association toward the null. Thus, the reported protective effects of IEM may be conservative. Future studies should consider multiple imputation approaches or, ideally, improved collection of parental education data in ageing cohorts to minimise this source of bias.

## CONCLUSIONS

We found IEM to be a powerful, independent determinant of IC globally. The findings underscore that education is not just a social ladder but a biological shield. For policymakers, the ‘Chinese exception’ (parallel ageing) highlights an urgent need for environmental and systemic interventions – improving air quality, healthcare equity, and social protection – to complement individual educational gains [33]. Furthermore, the dominance of the ‘direct effect’ suggests that educational policies focusing on cognitive development and early-life enrichment may yield greater healthy longevity dividends than purely economic transfers [34]. Promoting upward mobility, especially for socially disadvantaged groups, represents a viable strategy to narrow health inequalities and foster healthy ageing in an ageing world [12].

## Additional material

Online Supplementary Document

## Data Availability

**Data availability:** The data are available from the corresponding author upon reasonable request.
