# Supplementary material for: Intergenerational educational mobility and intrinsic capacity in later life: a harmonised analysis of individuals from five countries
Source: J Glob Health. 2026 Oct 9;16:04304. doi: 10.7189/jogh.16.04304 (PMC13650976; doi:10.7189/jogh.16.04304)
Supplement: Supplementary file 1 [file jogh-16-04304-s001.pdf]

Supplement to: Guo L, Wang H, Teng W, Bai B, Sang B, Zhang L. Intergenerational educational mobility and intrinsic capacity in later life: a harmonised analysis of individuals from five countries. J Glob Health. 2026;16:04304.

Figure S1. Spearman's rank correlation heatmap of variables in the pooled sample

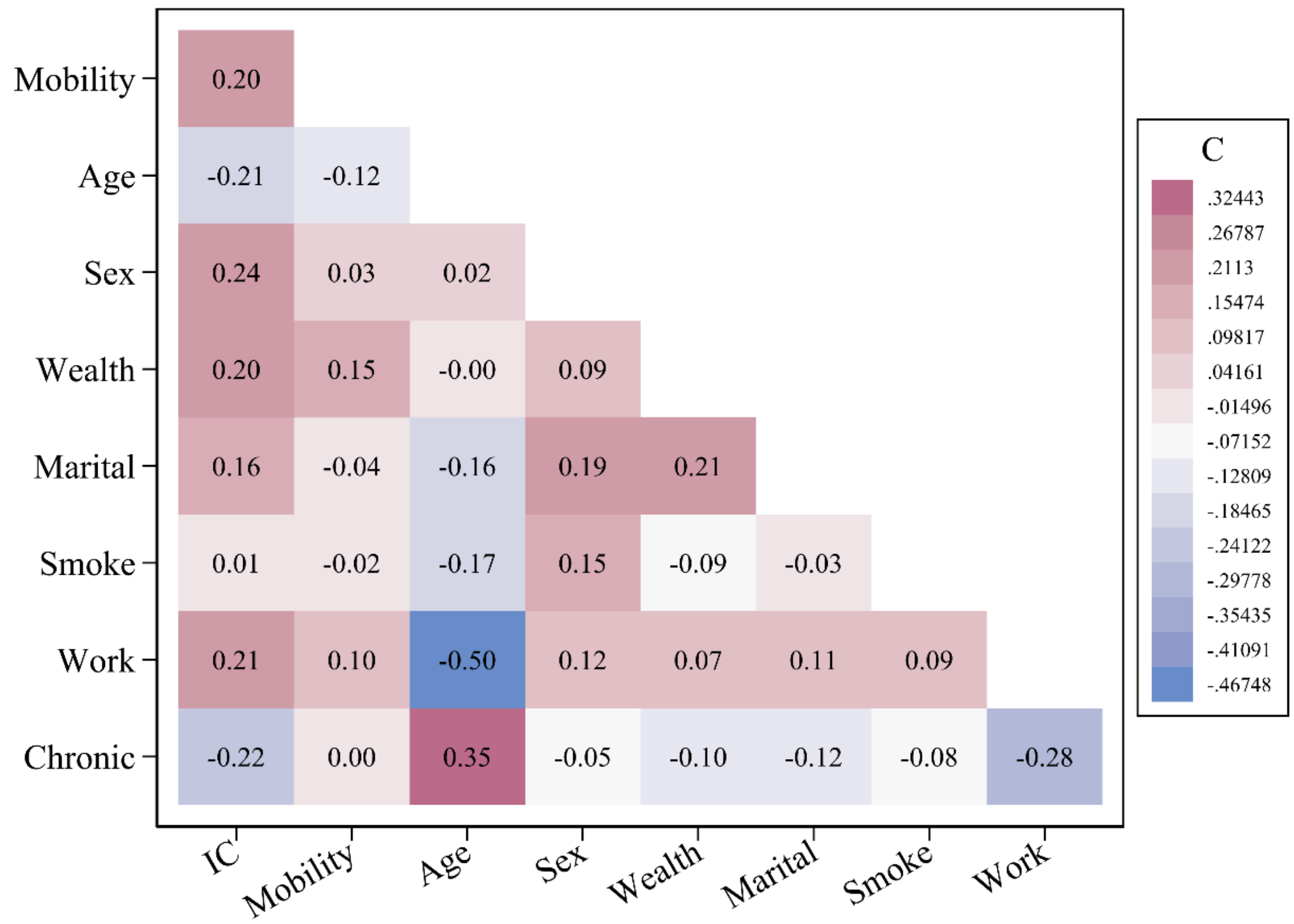

Note: Values in the grid represent Pearson correlation coefficients (r). Variable Definitions:IC (Intrinsic Capacity): A standardized composite score (Mean=0, SD=1) derived from the WHO framework, encompassing five domains: cognition, locomotion, vitality, sensory function, and psychological well-being. IEM: Categorized educational trajectory from parents to offspring based on ISCED-97 (1=Stable Low, 2=Upward Mobility, 3=Stable High, 4=Downward Mobility). Note: For correlation purposes, this ordinal coding reflects a gradient of educational accumulation. Age: Chronological age at interview (years). Sex: Binary variable (0=Female, 1=Male). Wealth: Household wealth tertiles (1=Bottom, 2=Middle, 3=Top). Marital: Marital status (1=Married/Partnered, 0=Others). Smoke: Smoking status (1=Current smoker, 0=Non-smoker). Work: Employment status (1=Currently working, 0=Not working). Chronic: Baseline health status measured by the cumulative count of seven major chronic conditions (hypertension, diabetes, cancer, lung disease, heart disease, stroke, arthritis).

Figure S2. Meta-analysis of the association between IEM and intrinsic capacity across five international cohorts

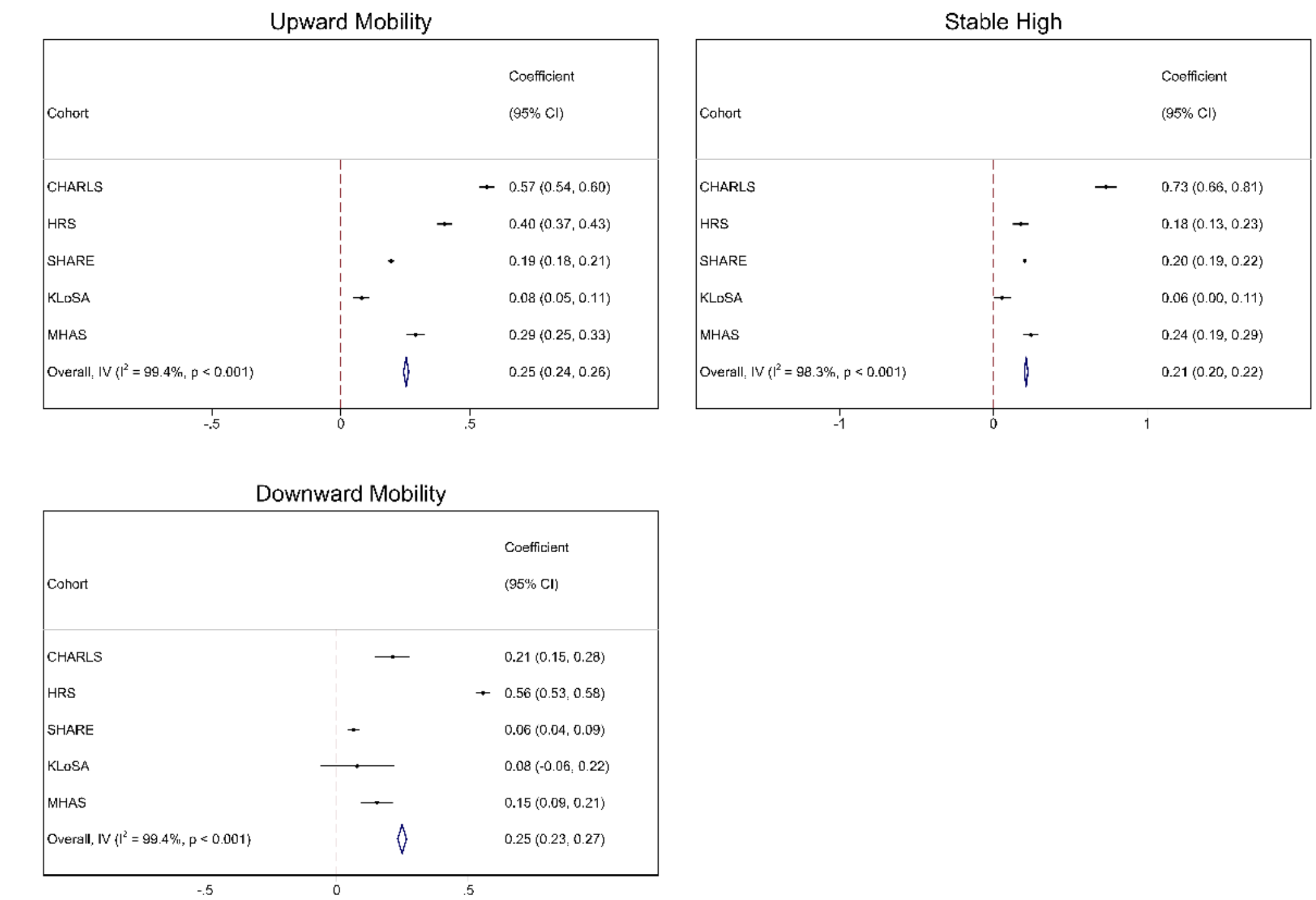

Note: Forest plots show pooled standardized coefficients (β) and 95% CIs for upward mobility, stable high education, and downward mobility, with "stable low" as the reference. Pooled estimates were calculated using random-effects models. Estimates for each cohort were adjusted for age, sex, marital status, household wealth, smoking, employment, chronic conditions, and survey waves. I^2 indicates heterogeneity. Abbreviations: CHARLS, China; HRS, USA; SHARE, Europe; KLoSA, South Korea; MHAS, Mexico.

Table S1. Detailed measurement and harmonization of study variables across five cohorts

**Selection Step**	**CHARLS**	**HRS**	**SHARE**	**KLOSA**	**MHAS**
Initial sample size (observations)	96628	208674	327266	44273	76506
**Excluded:**					
Age < 50 years or missing age	-10284	-6364	-6394	-271	-31548
Missing intrinsic capacity data	-5360	-27	-0	-0	-3438
Missing educational mobility data	-1492	-34317	-73917	-439	-4031
Missing covariates	-25639	-782	-53188	-734	-94
Final analytic sample	53853	167184	193767	42829	37395

Note: Covariates include demographic characteristics (age, sex, marital status), socioeconomic status (household wealth tertiles, employment), lifestyle behaviors (smoking status), and baseline health (chronic condition count).

Table S2. Detailed measurement and harmonization of study variables across five cohorts

Variable	General Definition & Harmonization	Cohort-Specific Measurement Details
**OUTCOME**	Intrinsic Capacity (IC)	Composite Z-score of 5 domains:
1. Cognition	Episodic Memory & Mental StatusSum of immediate recall, delayed recall, and mental status tests.	CHARLS, HRS, KLoSA, MHAS: Immediate/delayed word recall (10-word list) + Serial 7s subtraction or date naming.SHARE: Immediate/delayed word recall (10-word list) + Verbal fluency (animal naming).
2. Locomotion	Physical PerformanceObjective assessment of lower body function.	CHARLS, SHARE, MHAS: Walking speed (time to walk 2.5m or 3m) or Chair rise time (5 repetitions).HRS: Walking speed (2.5m) or full physical performance battery.KLoSA: Self-reported difficulty in climbing stairs (due to lack of objective tests in early waves).
3. Vitality	Handgrip StrengthMaximum value of two trials using a dynamometer (kg).	All Cohorts: Measured using a dynamometer. Values were standardized by sex and Body Mass Index (BMI) within each cohort to account for biological differences.
4. Sensory	Vision & HearingSelf-reported functional ability.	All Cohorts: 5-point Likert scale (Excellent to Poor). Harmonization: Binary or ordinal coding derived from questions: "Is your eyesight/hearing excellent, very good, good, fair, or poor?" (Reverse coded).
5. Psychological	Depressive SymptomsAbsence of depressive symptoms (Reverse coded).	HRS, CHARLS, KLoSA, MHAS: CES-D Scale (Center for Epidemiologic Studies Depression Scale). Typically short form (8-item or 10-item).SHARE: Euro-D Scale (12-item).Harmonization: All scales standardized to Z-scores to ensure comparability despite different instruments.
**EXPOSURE**	Educational Mobility	Comparing Offspring vs. Max(Father, Mother) Education
Education Level	Binary Coding (Low vs. High)Based on International Standard Classification of Education (ISCED-97).	Low Education: • CHARLS/MHAS/KLoSA: Primary school or below (reflecting developing contexts).• HRS/SHARE: Lower secondary or below.High Education: • CHARLS/MHAS/KLoSA: Middle school or higher.• HRS/SHARE: Upper secondary (High school) or tertiary.
Categories	4 Trajectories	1. Stable Low: Parent Low → Self Low2. Upward: Parent Low → Self High3. Stable High: Parent High → Self High4. Downward: Parent High → Self Low
**COVARIATES**		
Wealth	Household Wealth TertilesTotal assets (financial + housing + other real estate) minus debts.	All Cohorts: Calculated as the sum of all household component assets. Note: Wealth was stratified into tertiles (Bottom, Middle, Top) separately for each country and each wave to account for purchasing power parity (PPP) and inflation.
Chronic	Multimorbidity CountSum of doctor-diagnosed conditions (0-7).	All Cohorts: Hypertension, Diabetes, Cancer (excluding skin cancer), Chronic Lung Disease, Heart Disease, Stroke, Arthritis.
Demographics	Standard Demographic Indicators	Age: Continuous (centered at 60).Sex: Male vs. Female.Marital: Married/Partnered vs. Unmarried/Widowed/Divorced.
Lifestyle	Smoking & Employment	Smoking: Current smoker (Yes/No).Employment: Currently working for pay (Yes/No).

Table S3. Baseline Characteristics of the Included Analytic Sample vs. Excluded Sample

Characteristics	Included Analytic Sample (N = 495,028)	Excluded due to missing IEM (N = 114,198)	P-value
Age, years (Mean ± SD)	65.94 ± 9.72	70.30 ± 11.89	< 0.001
Sex, Male (%)	43.68%	42.78%	< 0.001
Household Wealth (%)			< 0.001
- Low Tertile	31.06%	41.78%	
- Middle Tertile	32.97%	33.47%	
- High Tertile	35.97%	24.75%	
Intrinsic Capacity Score (Mean ± SD)	0.035 ± 0.978	-0.187 ± 1.058	< 0.001

Note: *P*-values were derived from two-sample t-tests for continuous variables (Age, Intrinsic Capacity Score) and Pearson's chi-squared tests for categorical variables (Sex, Household Wealth). Intrinsic Capacity scores represent standardized Z-scores. The excluded sample represents individuals who met age criteria but lacked complete parental or personal educational data.
